# Transcriptional Suppression of Renal Antioxidant Enzyme Systems in Guinea Pigs Exposed to Polymerized Cell-Free Hemoglobin

**DOI:** 10.3390/toxics4010006

**Published:** 2016-02-19

**Authors:** Otgonchimeg Rentsendorj, Xiaoyuan Zhang, Matthew C. Williams, Paul W. Buehler, Felice D’Agnillo

**Affiliations:** Laboratory of Biochemistry and Vascular Biology, Division of Hematology Research and Review, Center for Biologics Evaluation and Research, Food and Drug Administration, Silver Spring, MD 20993, USA; otgonchimeg.rentsendorj@fda.hhs.gov (O.R.); xiaoyuan.zhang@fda.hhs.gov (X.Z.); matthew.williams@fda.hhs.gov (M.C.W.); paul.buehler@fda.hhs.gov (P.W.B.)

**Keywords:** hemoglobin-based oxygen carrier (HBOC), kidney, oxidative stress, antioxidants, DNA methylation

## Abstract

Hemoglobin-based oxygen carriers (HBOCs) are being developed as oxygen and plasma volume-expanding therapeutics though their potential to promote oxidative tissue injury has raised safety concerns. Using a guinea pig exchange transfusion model, we examined the effects of polymerized bovine hemoglobin (HbG) on the transcriptional regulation, activity, and expression of the renal antioxidant enzymes; superoxide dismutase (SOD), catalase (CAT), and glutathione peroxidase (GPx). HbG infusion downregulated the mRNA levels for genes encoding SOD isoforms 1-3, GPx1, GPx3, GPx4, and CAT. This transcriptional suppression correlated with decreased enzymatic activities for SOD, CAT, and GPx. Immunostaining revealed decreased protein expression of SOD1, CAT, and GPx1 primarily in renal cortical tubules. DNA methylation analyses identified CpG hypermethylation in the gene promoters for SOD1-3, GPx1, GPx3, and GPx4, suggesting an epigenetic-based mechanism underlying the observed gene repression. HbG also induced oxidative stress as evidenced by increased renal lipid peroxidation end-products and 4-HNE immunostaining, which could be the result of the depleted antioxidant defenses and/or serve as a trigger for increased DNA methylation. Together, these findings provide evidence that the renal exposure to HbG suppresses the function of major antioxidant defense systems which may have relevant implications for understanding the safety of hemoglobin-based products.

## 1. Introduction

Chemically modified or recombinant hemoglobin (Hb)-based oxygen carriers (HBOCs) are being developed as oxygen and volume replacement therapy for clinical indications such as emergency resuscitation, elective surgery, organ perfusion, and others [1,2]. HBOCs have several potential benefits over red blood cells (RBCs) including universal compatibility, immediate availability, and long-term storage. Despite these important advantages, HBOC development in the United States has been slowed by safety concerns prompted by reports of serious adverse events, including death, in late phase clinical trials [2,3]. While the exact mechanisms underlying these safety issues are not completely understood, the propensity for extracellular Hb to generate and propagate oxidative stress is thought to be an important contributing factor [1,2,3].

Outside the natural protective environment of the RBC, Hb can undergo uncontrolled redox transformations to non-functional and/or toxic protein-bound and unbound species including methemoglobin (ferric, Fe^3+^), ferryl heme intermediate (Fe^4+^), hemichromes, and heme or labile plasma iron that can initiate or propagate oxidative damage to lipids, nucleic acids, and proteins [1,2]. Reactive oxygen species (ROS) can trigger and sustain the redox reactions of Hb and its breakdown products ultimately leading to organ dysfunction or injury when protective mechanisms are diminished or overwhelmed. For example, we reported that polymerized Hb transfusion in guinea pigs triggers blood brain barrier (BBB) disruption characterized by alterations in cerebral endothelial tight junctions, increased BBB permeability, astrocyte and perivascular cell activation, and increased oxidative markers including heme oxygenase-1 (HO-1) and lipid peroxidation end products [4]. Renal exposure to oxidized human Hb also increased nuclear factor erythroid 2-derived-factor 2 (Nrf-2) and HO-1 expression, non-heme iron deposition, lipid peroxidation, interstitial inflammatory cell activation, and tubular and glomerular injury markers [5].

Oxidative stress is generated by an imbalance between ROS-producing systems and antioxidant defense mechanisms. Major antioxidant defense mechanisms include: (i) cytoplasmic CuZnSOD (SOD1), mitochondrial MnSOD (SOD2), extracellular SOD (EC-SOD or SOD3) and catalase (CAT), which collectively remove superoxide (●O_2_^−^) and hydrogen peroxide (H_2_O_2_); (ii) glutathione peroxidases (GPx) and glutathione reductase (GR) that reduce H_2_O_2_ to H_2_O; and (iii) ascorbate and vitamin E, which function to terminate lipid chain reactions involving peroxyl radicals [6]. Epigenetic modification of the genome by oxidative stress is thought to play a key role in altering antioxidant gene transcription and mRNA stability in diseases such as diabetes and cancer [7,8]. DNA hypermethylation, especially of CpG island sites in promoter regions, is considered an important modification that prevents transcription factor recruitment and consequently suppresses the transcription of various genes [9]. Many human cancers including breast cancer, prostate cancer, and lung carcinomas show reduced SOD and GPx activity that, in some cases, was linked to epigenetic mechanisms including DNA methylation [10,11,12,13,14,15]. Similarly, ROS-mediated CAT downregulation and methylation of its gene promoter were also confirmed during hepatocellular carcinoma development [16].

HBOCs can act as a source of and/or exacerbate ROS production, however little is known about whether HBOCs can influence the functional status of antioxidant defense systems. Using a guinea pig exchange transfusion (ET) model, we examined the effects of polymerized bovine hemoglobin (HbG) on the transcriptional regulation, activity, and expression of the renal antioxidant enzymes; superoxide dismutase (SOD), catalase (CAT), and glutathione peroxidase (GPx). Species such as guinea pigs are useful for examining Hb oxidative processes because, similar to humans, they lack the ability to produce ascorbate which is a powerful reductant capable of controlling intravascular Hb oxidation [17,18]. Using this model, we previously showed that HbG induced renal HO-1 and *L*-ferritin expression that was accompanied by significant iron deposition [19]. Here, we present evidence that HbG transfusion suppresses renal antioxidant enzyme expression at the gene and protein level, possibly through epigenetic alterations involving DNA methylation.

## 2. Materials and Methods

### 2.1. Chemicals and Antibodies

Oxyglobin (HbG) was purchased from Biopure Corporation (Cambridge, MA, USA). HbG is chemically modified hemoglobin solution containing a heterogeneous mixture of glutaraldehyde-polymerized bovine hemoglobin at a concentration of 13 g/dL in modified lactated Ringer’s. A detailed description of the physicochemical properties of the mixture as a whole and of each individual fraction has been described elsewhere [20]. Rabbit polyclonal antibodies to SOD1, GPx1, and CAT were purchased from Abcam (Cambridge, MA, USA). Mouse monoclonal 4-hydroxynonenal (4-HNE) antibody was purchased from Genox (Baltimore, MD, USA).

### 2.2. Surgical Preparation and Experimental Protocol

Male Hartley guinea pigs were purchased from Charles Rivers Laboratories (Wilmington, MA, USA) and acclimated for one week upon arrival at the FDA/CBER animal care facility. All animals were fed normal diets during the acclimation period and weighed 350–450 g at the time of study. Animal study protocol (ASP#2004-12, 2013) was approved by the FDA/CBER Institutional Animal Care and Use Committee with all experimental procedures performed in adherence to the National Institutes of Health guidelines on the use of experimental animals. Surgical preparation and catheter implantation was performed as previously described [17]. Twenty-four hours after recovery from surgical catheter implantation, fully conscious and freely moving guinea pigs underwent a 50% exchange transfusion (ET) replacing blood with HbG as previously reported [19]. Based on our previous studies with this model, the calculated exposure parameters of dose, maximum plasma concentration (Cmax), and area under the plasma concentration *versus* time curve (AUC_0-∞_) of HbG following transfusion were 3272 ± 106 mg/kg, 40.0 ± 2.22 mg/mL and 788.1 ± 90.6 mg·mL·h^−1^, respectively. These exposure parameters were associated with a circulatory half-life of 15.7 h [17]. Sham control animals underwent the surgical procedure and were allowed to recover for 24 h prior to sacrifice. At the indicated times, the animals were anesthetized, the femoral veins were cut, and cold saline was perfused via the arterial catheter to remove blood. Kidneys were dissected, cut in half, and frozen immediately in liquid nitrogen and stored at −80 °C or fixed in 10% formalin.

### 2.3. Design of PCR Primers and TaqMan Probes for Quantitative Gene Expression Assay

PCR primers and TaqMan MGB probes for amplification of guinea pig SOD1-3, GPx1, GPx3, and GPx4 were designed using the Primer Express 2.0 software (Applied Biosystems, Foster City, CA, USA, 2001). The mRNA sequences were obtained from NCBI GenBank, and the corresponding accession numbers for each gene of interest is provided in Table 1. The TaqMan gene expression assays for CAT and the housekeeping gene GAPDH for guinea pig were purchased from inventoried assays of Life Technologies (Carlsbad, CA, USA). The PCR primer and TaqMan probe sequences used to quantify the mRNA levels of the genes of interest are shown in Table 1.

### 2.4. RNA Extraction

Total RNA was extracted from kidney slices (transverse cut) using Trizol (Life Technologies, Carlsbad, CA, USA) and isolated with RNeasy Mini Kit (Qiagen, Germantown, MD, USA) following the manufacturer’s instructions. Genomic DNA was eliminated from the samples by the on-column digestion using RNase-free DNase treatment (Qiagen). RNA quality and quantity was assessed using a NanoDrop ND-1000 spectrophotometer. The integrity of total RNA was evaluated by ethidium bromide staining on denaturing agarose gels. The RNA was then stored at −80 °C before further processing.

### 2.5. Real-Time Quantitative-PCR (qPCR)

Purified total RNA (1 µg) was reverse-transcribed to cDNA with TaqMan Reverse Transcription Reagents using random hexamers (Life Technologies) in a 20 µL volume. One µL of each amplified cDNA of a 5× or 10× dilution was subjected to qPCR using TaqMan gene-specific forward and reverse primers and probes and the TaqMan Fast Universal PCR Master Mix (Life Technologies) in 10 μL reaction volumes in duplicate or triplicate. The PCR amplification of all transcripts was performed on the ABI ViiA7 machine (Applied Biosystems, Grand Island, NY, USA). The qPCR program used was: 95 °C for 10 min and 40 cycles of 95 °C for 15 s followed by 60 °C for 20 s. Expressions of each target gene were calculated by the delta-delta Ct method and normalized to GAPDH.

### 2.6. Antioxidant Enzyme Activity Assays

Colorimetric assay kits for measuring total SOD, GPx, and CAT activity in renal tissue homogenates were purchased from Abcam (Cambridge, MA, USA) and performed according to manufacturer’s instructions. Enzyme activities were expressed as U/mg protein.

### 2.7. Immunohistochemistry

Paraffin-embedded 5 µm sections were dewaxed, rehydrated, and heat-treated in a microwave oven for 15 min in 10 mM sodium citrate buffer, pH 6.0. After cooling for 30 min at room temperature, slides were washed in phosphate-buffered saline with 0.05% Tween-20 (PBST) and incubated with Bloxall (Vector Laboratories, Burlingame, CA, USA) for 15 min at room temperature to inhibit endogenous peroxidases. After a brief wash, sections were blocked in 2.5% horse serum for 30 min at room temperature and then incubated overnight at 4 °C with antibodies to SOD1 (1:1000), GPx1 (1:100), CAT (1:2000), and 4-HNE (1:500) in PBST containing 2.5% horse serum. Signal was developed using polymeric peroxidase-conjugated secondary antibodies (Vector ImmPRESS Kit) and DAB (Vector Laboratories, Burlingame, CA, USA). Slides were then dehydrated in graded ethanol and SafeClear, and mounted using Permount. Images were scanned using a NanoZoomer 2.0-RS Digital slide scanner (Hamamatsu Corporation, Bridgewater, NJ, USA) and processed with NDP view2 software (Ver. 2.5/Rev.1, Hamamatsu Corporation, 2015).

### 2.8. CpG Island Prediction and Primer Design for Promoter Methylation PCR

The genomic DNA sequences of guinea pig SOD1-3, CAT, GPx1, GPx3, and GPx4 were obtained from NCBI GenBank (Genome accession numbers are listed in Table 2). The promoter methylation regions were identified by MethPrimer [21] and CpG Island Searcher [22]. The MseI endonuclease restriction maps were determined using NEBcutter V2.0 software (Oxford University Press, Oxford, UK, 2003) [23]. Target-specific PCR primers located within the two MseI restriction sites (TTAA) that correspond to the CpG island were designed using Primer-BLAST [24] online software (Table 2). A separate BLAST search for each primer was performed to ensure that the primer did not recognize unintended targets [25].

### 2.9. Analysis of DNA Methylation

Genomic DNA was isolated from transverse cut kidney slices using PrepEase genomic DNA isolation kit from Affymetrix (Santa Clara, CA, USA). DNA quality and quantity were assessed using a NanoDrop ND-1000 spectrophotometer. Methylation status of the antioxidant enzyme promoter regions was determined using a Promoter Methylation PCR Kit purchased from Affymetrix following the manufacturer’s instructions (Santa Clara, CA, USA). Briefly, 5 µg genomic DNA samples (the genomic DNA derived from three animals was pooled and used as a template/group) were digested with MseI restriction enzyme (New England Biolabs, Ipswich, MA, USA) for 2 h at 37 °C in a 30 μL volume to produce small fragments of DNA that retain the CpG islands. To verify DNA fragmentation, 500 ng samples were run on a 0.7% agarose gel for 2 h at 100 V in 1X TBE buffer, and visualized by ethidium bromide staining. Fragmented DNAs were purified using DNA purification spin columns (kit supplied) and eluted in 10 μL water. Purified DNA fragments were then incubated with a MeCP2 (a methylation binding protein or MBP) on ice for 30 min to form a MBP/DNA complex and the entire complex was then transferred to a Separation Spin Column. MBP/DNA complexes were separated from free DNA fragments by washing three times with ice-cold Column Wash Buffer and the flow-through containing free non-methylated DNA fragments was discarded. Column trapped methylated genomic DNA fragments were then eluted in 10 μL Column Elution Buffer in a clean 1.5 mL microcentrifuge tube. One μL eluted DNA was used as a template in 20 μL reaction volume and was subsequently amplified by PCR using Platinum Taq DNA Polymerase (Life Technologies). The amplification conditions were as follows: 2 min denaturation at 95 °C ; 40 cycles at 95 °C for 15 s, at 58 °C for 20 s, and at 72 °C for 20 s; followed by 7 min at 72 °C. PCR products were loaded onto 2% low melting agarose gel and separated by electrophoresis, and were visualized by ethidium bromide staining.

### 2.10. Lipid Peroxidation Assay

Lipid peroxidation was assessed using a colorimetric assay kit that measures free malondialdehyde (MDA) and 4-hydroxyalkenals (HAE) as decomposition products of lipid peroxides (Bioxytech LPO-586, Oxis International, Portland, OR, USA). Kidney homogenates were prepared using Tris buffer pH 7.4 containing 5 mM butylated hydroxytoluene (BHT) to avoid additional peroxidation during the homogenization process. Standards were prepared using 1,1,3,3 –tetramethoxypropane (TMOP) and values were calculated as pmol/mg kidney protein.

### 2.11. Statistical Analysis

Data are represented as means ± standard error (SE) for replicate experiments (3–5 animals per group). Statistical analysis was performed by Student’s *t*-test (two-sample, assuming unequal variances). *p* < 0.05 was considered statistically significant.

## 3. Results

### 3.1. Reduced Renal SOD, GPx, and CAT Transcription, Expression, and Activity Following HbG Infusion

**Figure 1:**
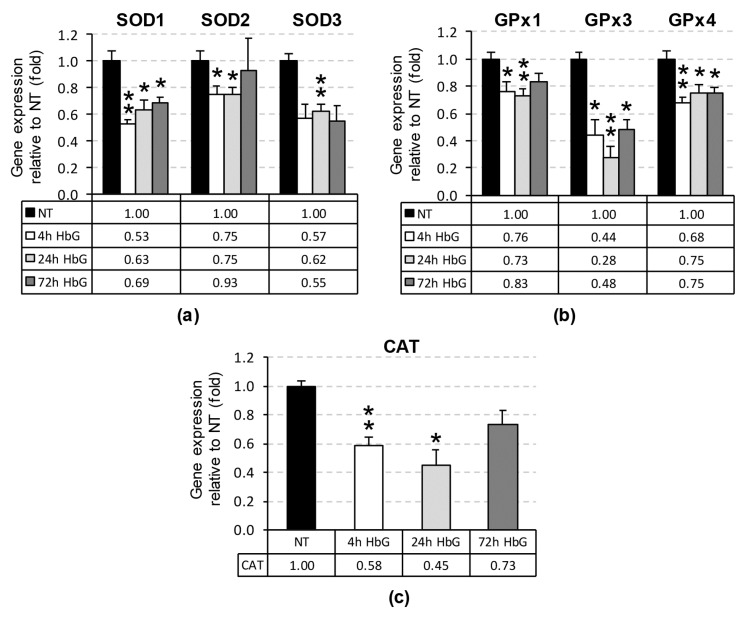
Renal antioxidant gene mRNA levels following infusion with HbG. The relative mRNA levels of SOD1-3 (**a**); GPx1, GPx3, and GPx4 (**b**); and CAT (**c**) were assessed by qPCR as described in “Materials and methods” after 4, 24, and 72 h post-ET. The reference value reported as fold change normalized to GAPDH and relative to NT, equals 1. Values are means ± standard error (SE) from 3 to 5 animals per group. Asterisks indicate significant differences compared to NT (* *p* < 0.05, ** *p* < 0.01). NT: sham-operated no treatment group.

Renal exposure to HbG has been shown to activate heme catabolic and iron sequestration pathways (*i.e.*, heme oxygenase, ferritin) [19]; however, the effects of HbG on antioxidant enzyme defenses are not well characterized. To determine the effect of HbG on the renal antioxidant enzyme response, we measured the transcript levels of major antioxidant defense genes including SOD1-3, GPx1, GPx3, GPx4, and CAT by real-time qPCR. The qPCR assay primer and probe sequences are listed in Table 1. Compared to sham controls, HbG downregulated the transcript levels for each of these antioxidant genes at 4, 24, and 72 h post-HbG infusion (*p* < 0.05) with the exception of CAT and SOD2 at the 72 h time point (Figure 1).

**Figure 2:**
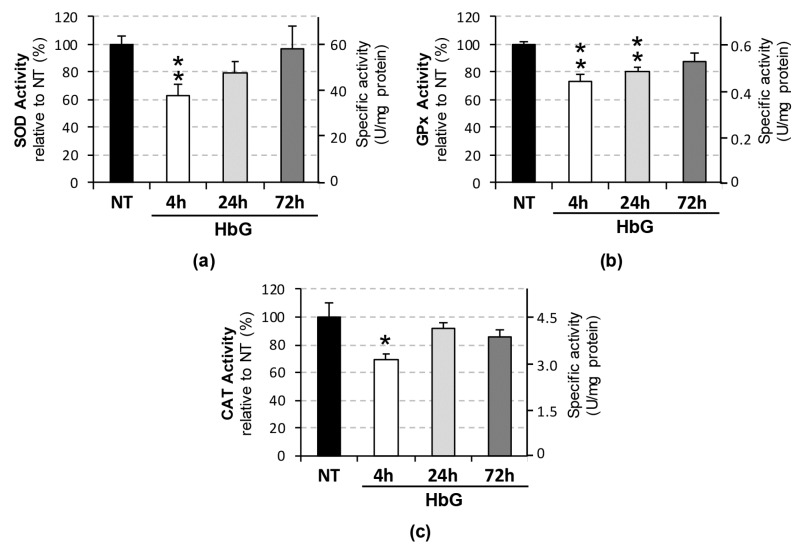
Renal antioxidant enzyme activities following infusion with HbG. Total SOD (**a**), GPx (**b**), and CAT (**c**) activities were measured at 4, 24, 72 h post-ET with HbG. Data are presented as percentage activities relative to NT (left axis) and estimated specific activities in U/mg protein (right axis). Values are calculated as the means ± SE for replicate experiments (*n* = 3–5 animals per group). Asterisks indicate significant differences compared to NT (* *p* < 0.05, ** *p* < 0.01).

To determine the effect of HbG on these antioxidant systems at the protein level, we measured the total enzymatic activities of SOD, GPx, and CAT in renal tissue homogenates (Figure 2). HbG produced significant decreases in the activities of SOD (62% ± 8.3%, *p* = 0.01), GPx (73% ± 5.4%, *p* = 0.009), and CAT (69% ± 4.4%, *p* = 0.041) at 4 h post-HbG infusion. SOD and CAT activities appeared to recover at 24 and 72 h, while decreased GPx activity was still noted at 24 h post-HbG (80% ± 3.3%, *p* = 0.002) but not at 72 h (Figure 2).

**Figure 3:**
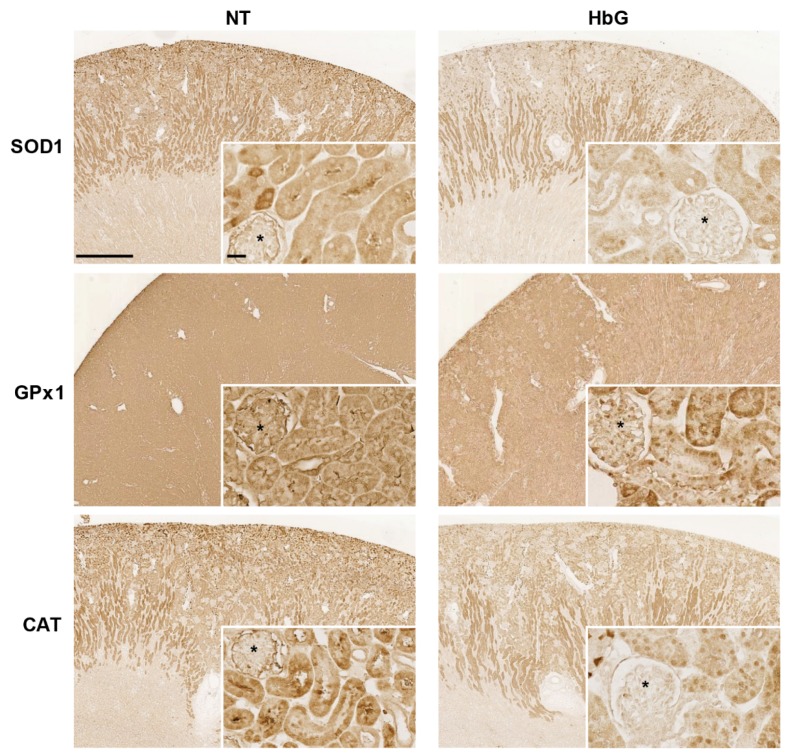
Immunohistochemical analysis of SOD1, GPx1, and CAT protein expression. Kidney sections from sham controls and 4 h (SOD1 and CAT) or 12 h (GPx1) post-ET with HbG were probed as described in the Materials and Methods. Reduced immunoreactivity (brown signal) was observed for all three enzymes in the HbG group compared to NT. Asterisks denote glomeruli. Scale bars: 1 mm (main panels) or 25 µm (inserts).

Next, we evaluated protein expression of some antioxidant enzymes, including SOD1, GPx1, and CAT by immunohistochemistry (Figure 3). Reduced immunoreactivity for SOD1 and CAT was observed as early as 4 h post HbG, while decreased GPx1 expression was evident at 12 h compared to sham controls. For all three enzymes, the reduced expression was particularly evident in the renal cortical tubules, and variable reduction was noted in glomeruli (Figure 3). Together, these results suggest that HbG disrupts the function of renal antioxidant enzyme defenses by a mechanism that involves the suppression of antioxidant gene transcription.

### 3.2. DNA Promoter Analysis: HbG-Transfusion Induces DNA Hypermethylation of SOD, GPx, and CAT

**Figure 4:**
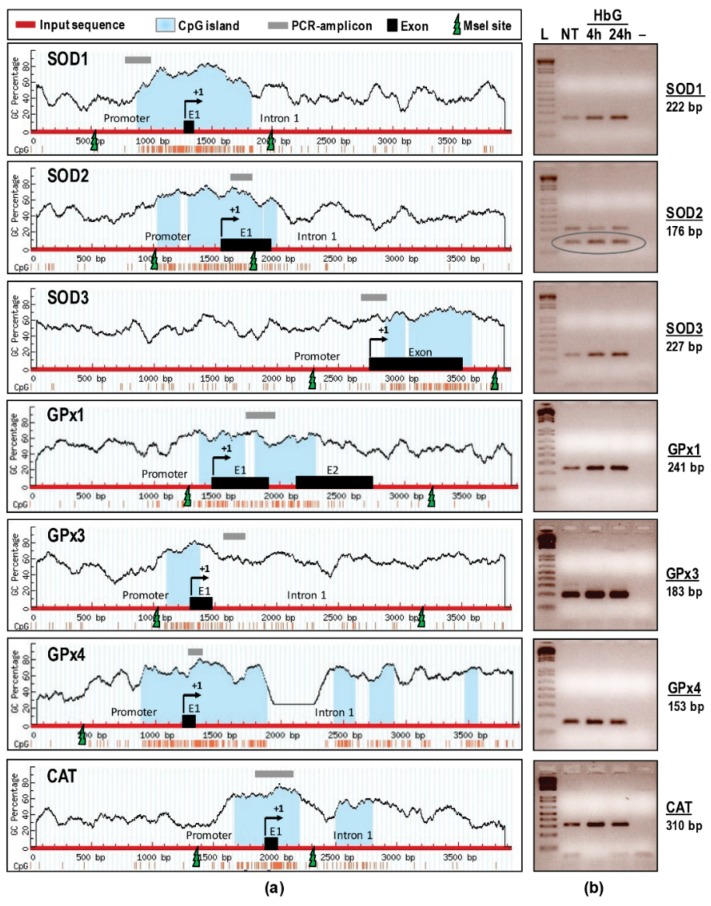
DNA methylation status of SOD1-3, GPx1, GPx3, GPx4, and CAT following HbG infusion. (**a**) CpG island search and restriction mapping. Four kb genomic regions (thick red line) harboring putative promoter, exon 1, and intronic regions of SOD1-3, GPx1, GPx3, GPx4, and CAT were subjected to sequence analysis. Located CpG islands indicated in light blue, PCR amplicons in grey line, exons (E1-E2) in black boxes, MseI restriction sites in green lightning bolts, and TIS labelled as +1. Vertical lines depict GC percentage; (**b**) Agarose gel images (inverted) of hypermethylated SOD1-3, GPx1, GPx3, GPx4, and CAT following HbG infusion. The expected sizes (in bp) of PCR amplicons listed on the right. L: Ladder (1 Kb Plus), NT: non-treatment group, HbG (4 h or 24 h): oxyglobin-infused groups at different time points. “−“: Template-less negative control. The SOD2 resulted in two distinct amplicons (approximately 176 bp and 310 bp), however the lower band with predominant intensity is the expected one (circled).

Emerging evidence suggests that DNA hypermethylation of CpG islands in transcriptional promoters can play an important repressive role in the oxidative stress response [9]. Using the MethPrimer and CpG Island Searcher programs, CpG islands (shaded in light blue) were determined for all antioxidants including SOD1-3, GPx1, GPx3, GPx4, and CAT (Figure 4a). The guinea pig genomic DNA sequences were obtained from NCBI GenBank (genome accession numbers are listed in Table 2). In the present study, we define the translation initiation site (TIS) as position “+1” to avoid the lengthy corresponding nucleotide positions in genomic sequences. Approximately 4 kb genomic DNA of SOD1 (in between −1277 and +2705), SOD2 (−1570 and +2332), SOD3 (−2773 and +1120), GPx1 (−1484 and +2417), GPx3 (−1306 and +2595), GPx4 (−1211 and +1790) and CAT (−1912 and +2019) were probed for in the computer simulations. Both programs we used returned similar results, however, the result of MethPrimer is shown for simplicity (Figure 4a). Analysis of the promoter region of different antioxidant genes revealed the presence of promoter CpG islands or “promoter hypermethylation region” in all the enzymes except for SOD3. However, for SOD3, the CpG island was still detected within an existing single exon with a GC content of 60% or more. A CpG island is defined by a CpG dinucleotide content of greater than 60%. Interestingly, a total of 1–4 CpG islands were detected in all antioxidant enzymes surrounding TIS, some of which were covering an entire exon 1 (E1) and part of the first introns (Figure 4a).

The methylation statuses of predicted CpG islands were further verified by Promoter Methylation PCR assay. We analyzed the SOD1-3, GPx1, GPx3, GPx4, and CAT methylations in sham controls and 4 and 24 h post-HbG renal samples. Agarose gel electrophoresis of all PCR amplicons showed single bands of correct sizes, except for SOD2 (Figure 4b). The SOD2 showed two distinct bands (176 bp and 310 bp) of which the lower band is the expected one (circled). In sham controls, the CpG islands for most of these genes showed low level methylation except for the higher methylation rate of the GPx3 gene. Compared to respective sham controls, HbG induced extensive hypermethylation as evidenced by darker and broader PCR bands for all the analyzed gene promoters at 4 and 24 h. These data support the idea that HbG-mediated downregulation of antioxidant gene transcription may be related to increased DNA hypermethylation of antioxidant gene promoter regions.

### 3.3. Increased Renal Lipid Peroxidation Following HbG Transfusion

**Figure 5:**
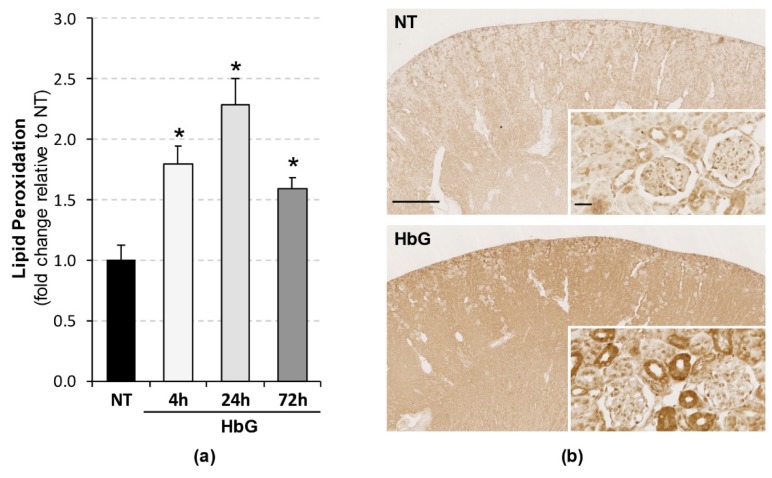
Renal lipid peroxidation following HbG infusion. (**a**) MDA+HAE levels were analyzed in renal tissue homogenates as described in the Materials and Methods. Results were calculated as pmol/mg protein and presented as the fold change relative to sham controls. Data are shown as the means ± SE for at least four animals per time interval. * *p* < 0.05 *versus* NT; (**b**) 4-HNE immunohistochemistry showing enhanced immunoreactivity (brown signal) in HbG group (4 h post-ET) compared to NT. Scale bars: 1 mm (main panels) or 25 µm (inserts).

Studies have shown that ROS catalyze DNA methylation [8,11,26]. To determine whether HbG induces renal oxidative stress, we measured the levels of free non-protein bound MDA and HAE as an indirect index of lipid peroxidation. HbG produced time-dependent increases in peroxidation end products relative to sham controls with peak levels attained at 24 h (Figure 5a). To more specifically assess the nature and localization of oxidative end products, we analyzed the accumulation of 4-HNE-modified protein adducts by immunohistochemistry. HbG enhanced 4-HNE immunoreactivity in the renal cortex characterized by a diffuse cytoplasmic localization pattern observed mainly in renal tubular cells (Figure 5b). These results confirm that HbG induces renal oxidative stress, which may play a role in the observed hypermethylation.

## 4. Discussion

The present study examined the transcriptional regulation, activity, and expression of cellular antioxidant systems in the kidney of guinea pigs transfused with HbG. The main findings were (i) HbG reduced the transcript levels of genes encoding for SOD (isoforms 1, 2, and 3), CAT, and GPx (isoforms 1, 3, and 4); (ii) HbG also decreased renal SOD, CAT, and GPx activity and protein expression of SOD1, CAT, and GPx1; (iii) HbG increased DNA methylation of CpG islands in the promoter regions of these antioxidant genes suggesting a possible epigenetic mechanism of gene repression; and (iv) HbG induced renal oxidative stress as evidenced by the accumulation of lipid peroxidation products and increased 4-HNE immunoreactivity. Consistent with these oxidative stress indices, we previously reported increased renal HO-1 expression, an important surrogate marker of oxidative stress, and significant non-heme iron deposition in HbG-infused guinea pigs [19]. Taken together, these findings suggest that modified Hbs, in addition to their ability to generate or promote ROS production, may influence oxidative stress by acting as a suppressor of antioxidant enzyme gene expression.

Free native Hb can readily dimerize from its tetrameric conformation and be filtered by renal glomeruli [2,27]. Filtered Hb is either taken up by proximal tubules via megalin and cubilin receptors or excreted in the urine when levels exceed reabsorption capacity. Consistent with renal promixal tubules being the site of Hb uptake, our immunohistochemical analyses showed that the loss of antioxidant protein expression and the increased 4-HNE immunoreactivity localized predominately to renal proximal tubules. Hb-induced renal dysfunction is thought to arise from tubular obstruction caused by precipitated Hb, ischemic injury, and/or heme iron-driven oxidative processes [2,27]. The nephrotoxicity associated with early generation HBOCs was partly attributed to the presence of unmodified Hb in these preparations [1,2]. Chemical and recombinant techniques to intramolecularly crosslink Hb as well as polymerization/conjugation techniques to further increase native Hb molecular size have improved the renal safety profiles of current generation HBOCs. With respect to the present model, it is important to highlight that HbG contains an unpolymerized Hb fraction of about 5% (compared to less than 1% for some of the HBOCs used clinically) which suggests a significant renal tubular exposure to filtered Hb [19]. Other design strategies have also been proposed to address the oxidative reactivity of HBOCs such as crosslinking Hb with GSH or SOD/CAT, or re-engineering the Hb molecule with favorable amino acid mutations [28,29,30]. Clearly, it will be important to compare the results obtained with HbG to other modified Hb preparations given that different HBOCs can exhibit different properties in terms of their oxygen affinity, molecular size, ability to extravasate, and/or oxidative stability.

Reduced functionality of enzymatic antioxidant defense systems has been implicated in many types of cancers [31], ischemia reperfusion [32,33], aging [34], and other chronic conditions like diabetes [35], obesity [36], and hypertension [37]. Exacerbation of hypoxic injury after reoxygenation is a key mechanism mediating injury in organ transplantation as well as myocardial, hepatic, gastrointestinal, cerebral, renal, and other ischemic syndromes. For instance, using a rat model of renal ischemia-reperfusion injury, Aragno *et al.* demonstrated renal ROS accumulation and loss of SOD, GPx, and CAT activity, and treatment with the antioxidant dehydroepiandrosterone attenuated renal injury [32]. While many studies have proposed a link between oxidative stress and antioxidant enzyme suppression in diverse pathological conditions, the molecular mechanisms underlying this event are not completely defined. Increasing evidence supports the concept that redox signaling and oxidative stress can affect the epigenetic regulation of genes, for example DNA hypermethylation and histone hypoacetylation at the genomic level [26]. Although DNA methylation is the best studied epigenetic mechanism, most recent studies on gene promoter methylation have focused on cancer-related genes [7,8]. Promoter methylation primarily inhibits gene expression through heterochromatin formation directly interfering with the binding of transcription factors to their target sites. For example, it is now firmly established that promoter hypermethylation is an important mechanism for tumor suppressor gene inactivation [8].

The linkage of a cytosine and guanine by a single phosphate forms a CpG dinucleotide complex in genomic DNA. DNA methylation occurs most commonly in CpG islands, which are often located in the promoter or first exon [38]. The common definition of a CpG island is a DNA stretch at least 200 bp long with a GC percentage greater than 60% [39]. Using two different computer simulation analyses, we detected putative CpG islands in all the antioxidant genes studied. A number of CpG island clusters (1–4) were located in close proximity to each other. Studies by others have shown that each CpG island may have its own specific function, and only a few CpG sites within each island may be critical for regulation [40]. This suggests that methylation of every site is not required for the regulation of gene expression. Our promoter methylation PCR assays further showed the existence of hypermethylated CpG islands within genes encoding SOD1-3, GPx1, GPx3, GPx4, and CAT in the HbG-infused animals. These results suggest that the CpG islands may be key regions involved in the regulation of renal antioxidant enzymes when subjected to HbG-induced oxidative stress. DNA methylation involves the conversion of cytosines into methylcytosines by DNA methyltransferases (DNMTs). The DNMT family includes DNMT1, which is predominantly responsible for CpG methylation, and DNMT3A and DNMT3B which are critical for *de novo* methylation. Given that oxidative stress has been shown to up-regulate DNMT activity and expression [41,42,43], we are currently examining whether HbG-mediated oxidative stress influences DNMT function as a mechanism to explain the observed DNA hypermethylation.

Previous studies showed that plasma and tissue antioxidant status play a critical role in controlling the redox activity and breakdown of HBOCs, which in turn can directly impact oxidative stress and heme handling pathways [17,19]. HbG was shown to oxidize more readily in the circulation of guinea pigs, a non-ascorbate-producing species with similar plasma and tissue antioxidant status to humans, compared to rats, an ascorbate-producing species [17]. Further comparative analyses in rats and guinea pigs showed that differing endogenous reductive capabilities can also impact the activation of heme catabolic and iron sequestration systems following HBOC infusion [19]. These observations suggest that preclinical testing using animal species with antioxidant capabilities similar to that of humans may be more predictive of the tissue response to HBOCs. The latter findings together with the present data also raise the important safety implication that individuals with preexisting antioxidant deficiencies associated with conditions such as diabetes, sepsis, and ischemia could be more prone to the adverse effects of HBOCs.

## 5. Conclusions

In the present study, a vital interplay between renal oxidative stress and epigenetic changes (CpG methylation) was identified in a guinea pig model of 50% ET with HbG. The finding that HbG alters the overall genomic and epigenetic regulation of enzymatic antioxidants provides new insight into molecular mechanisms that may contribute to the adverse effects of Hb-based therapeutics.

## Figures and Tables

**Table 1:** Taqman Assay Primers and Probes.

Gene	Accession Number	Forward Primer Sequence	Reverse Primer Sequence	Probes
SOD1	XM_003467248.3	GAGACCTGGGCAATGTGACT	GAGAGTCCTCGATGGATACATTGG	CACGCCGTCTGCACCAG
SOD2	XM_003466367.3	GACAAACCTGAGCCCTAATGGT	AGTCACGTTTGATGGCTTCCA	TTCCCCTTTGGGTTCTC
SOD3	XM_003467399.3	GCCGCGTCTGGAAACAC	CCTCGCCGGCATGGA	CCGCACTCGATCCTG
GPx1	XM_003476448.3	CTTCCCGTGCAACCAGTTC	CTTGAGCGAATGCAGAATCTCTTC	ACACCAGGAGAACGCC
GPx3	XM_003464498.3	GCGAGGAGTACATTCCCTTCAATAA	GCTGGCCACGTTGACAAAA	ACAGAGGCAAATACATCC
GPx4	NM_001256319.1	CCTTCCCCTGCAACCAGTT	GAACTCCTTGATCTCCTCATTGGT	CCTGGCTCCTGCTTCC
CAT	NM_001172925.1	Applied Biosystems	Cp03755233_m1	-
GAPDH	NM_001172951.1	Applied Biosystems	Cp03755743_g1	-

Sequences are given from 5′ → 3′. All probes were labeled with FAM™ dye.

**Table 2:** Primers used for DNA Methylation Analysis.

Genome	Accession Number	Forward Primer Sequence	Reverse Primer Sequence	Tm (°C)	Size (bp)
SOD1	NT_176367.1	CATCCATCTTGATGGGTCCT	GCGCCGGACTCGATTTA	57	222
SOD2	NT_176359.1	GGATCCCTGGGGTGATGTT	GCCCCATAGTCGTAGGGTAA	58	172
SOD3	NT_176369.1	GGATGAGGTGGATCTGTCG	GGCATACATGTCTCCGATCT	57	227
GPx1	NT_176411.1	CGAGAAGTTGGGGAGGAGTA	CGGACGTACTTGAGCGAATG	57	241
GPx3	NT_176338.1	TTCCACCAAAAGTGGGGGTC	GGCCTCAAGCAAGTCTTGGA	60	183
GPx4	NT_176099.1	ACCCCTGGCTTGATCGGAAC	CAGCTTCCAGGCACGGTCA	61	153
CAT	NT_176327.1	GCAGAGTTTGAAGTCGCCTA	ACCTAAAACGCCACACAAAC	57	310

Sequences are given from 5′ → 3′. The optimal melting temperature (Tm) is indicated for each primer pair. Expected lengths of the PCR products are shown in base pairs (bp).

## Abbreviations

The following abbreviations are used in this manuscript:

HBOC: Hemoglobin-based oxygen carrier
HbG: Oxyglobin/polymerized bovine hemoglobin
ET: Exchange transfusion
ROS: Reactive oxygen species
SOD: Superoxide dismutase
GPx: Glutathione peroxidase
CAT: Catalase
NT: No treatment
4-HNE: 4-hydroxynonenal
bp: Base pairs
